# Supplementary material for: Synthesis and Characterization of Imidazolium-Based Ionenes
Source: Molecules. 2025 Oct 2;30(19):3961. doi: 10.3390/molecules30193961 (PMC12525572; doi:10.3390/molecules30193961)
Supplement: Supplementary file 1 [file molecules-30-03961-s001.zip › molecules-3884793-supplementary.pdf]

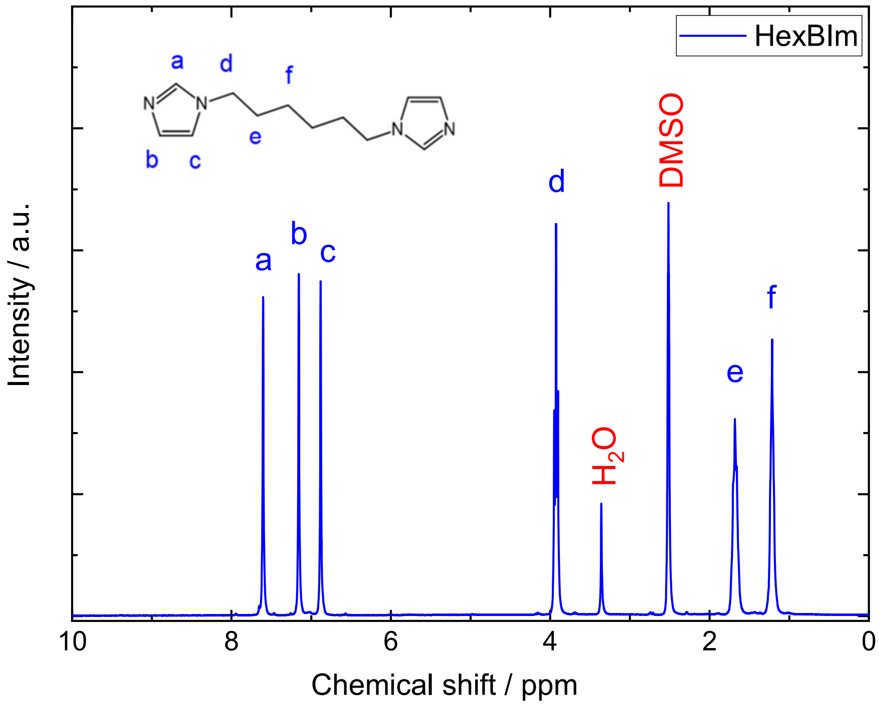

Figure S1: ^{1}H-NMR spectrum of **M1**

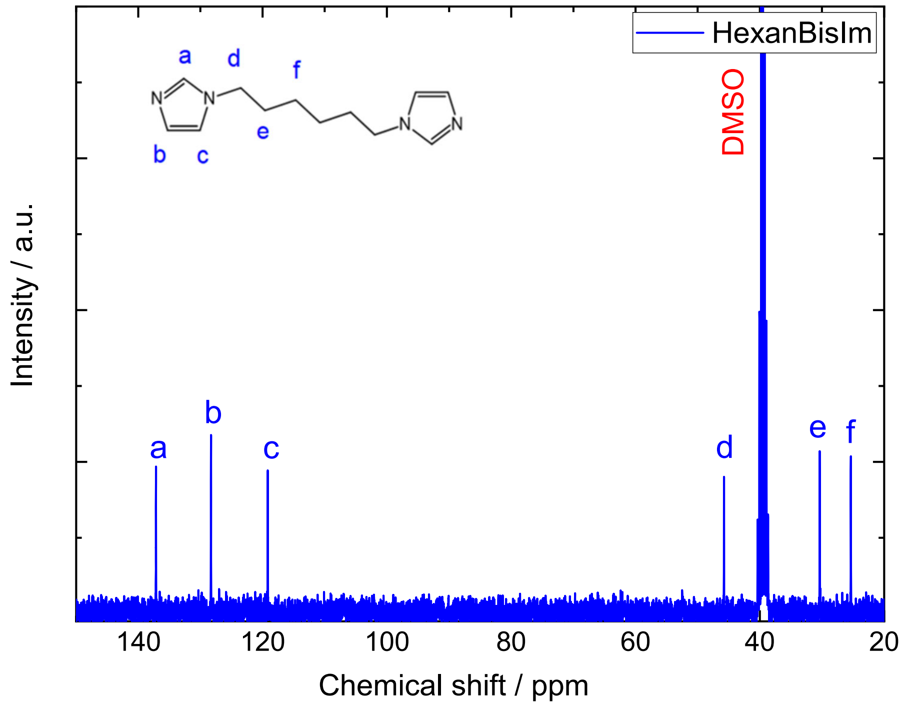

Figure S2: ^{13}C-NMR spectrum of **M1**

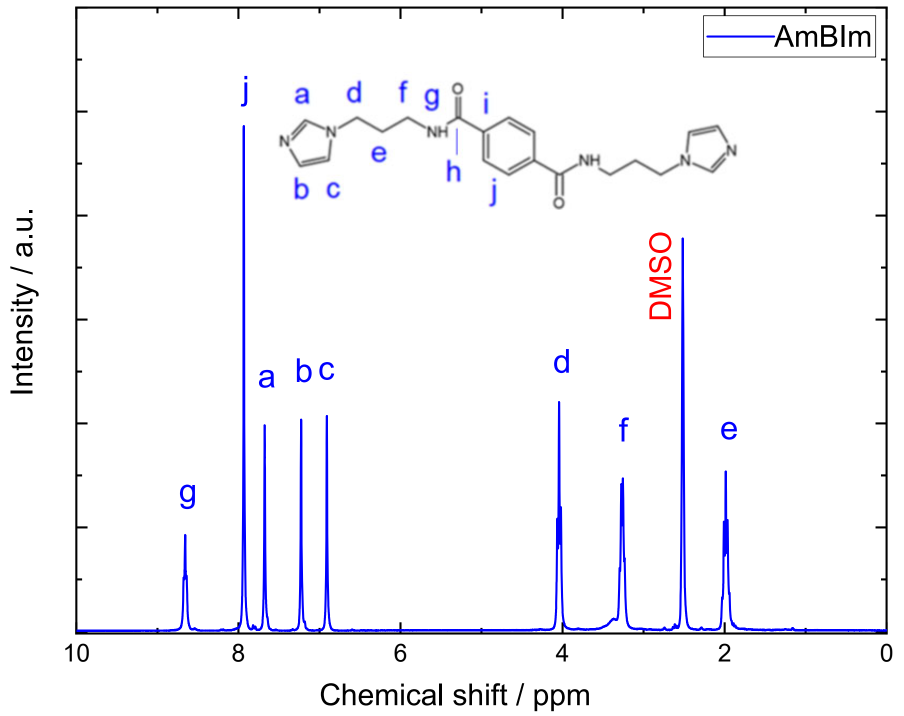

Figure S3: ^{1}H-NMR spectrum of **M2**

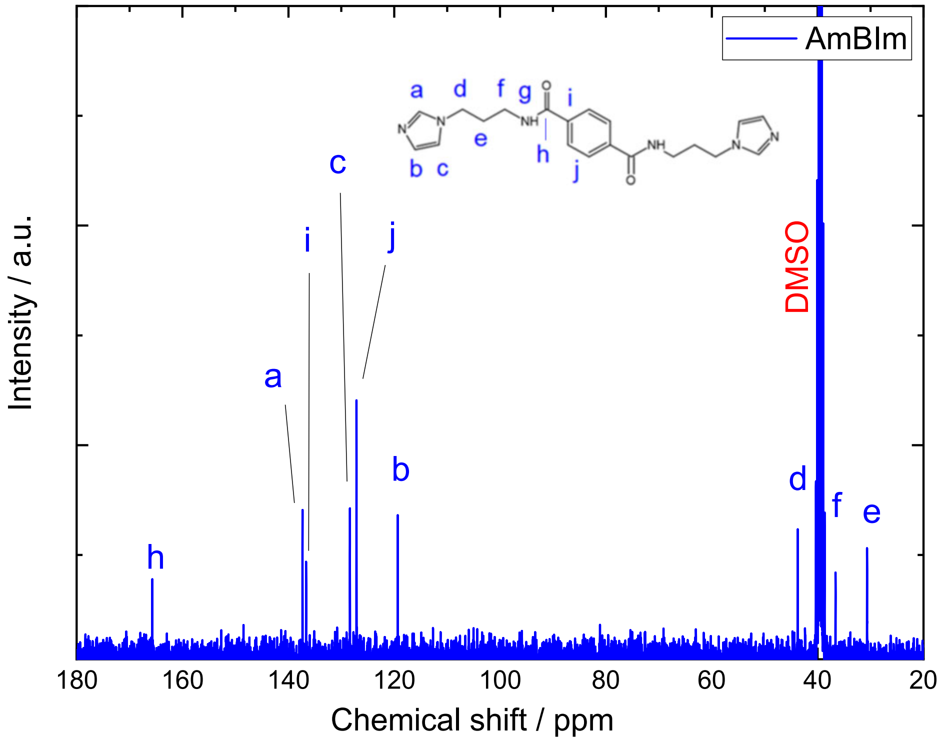

Figure S4: ^{13}C-NMR spectrum of **M2**

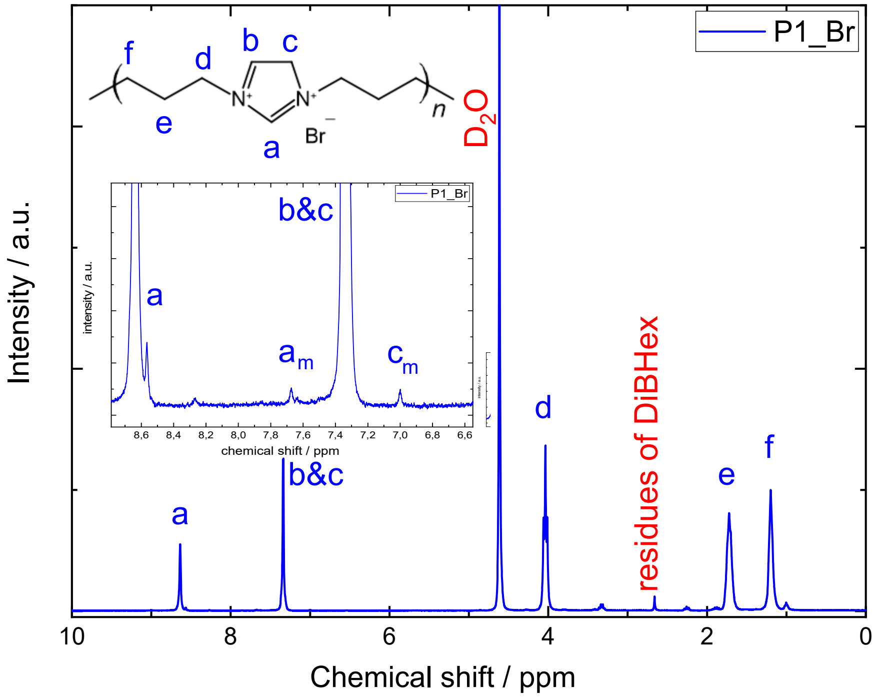

Figure S5: ^{1}H-NMR spectrum of **I1.** The signals (a_m), and (c_m) correspond to the protons of the imidazole end-groups.

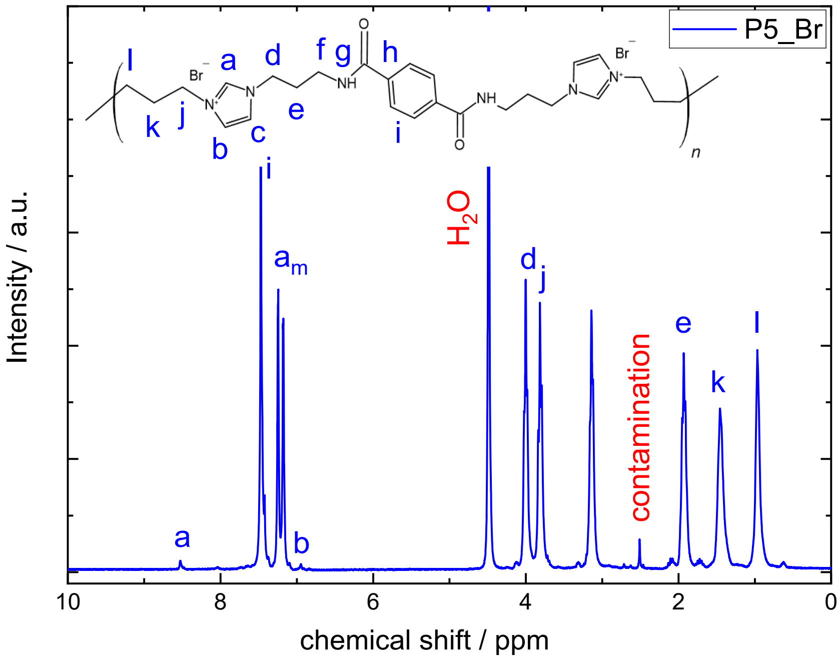

Figure S6: ^{1}H-NMR spectrum of **I2**

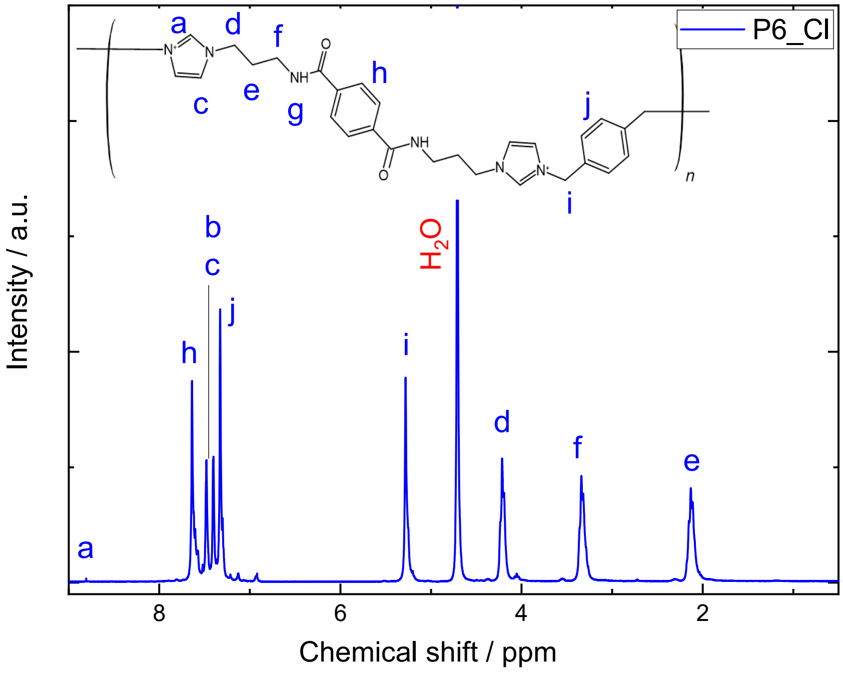

Figure S7: ^{1}H-NMR spectrum of **I3**

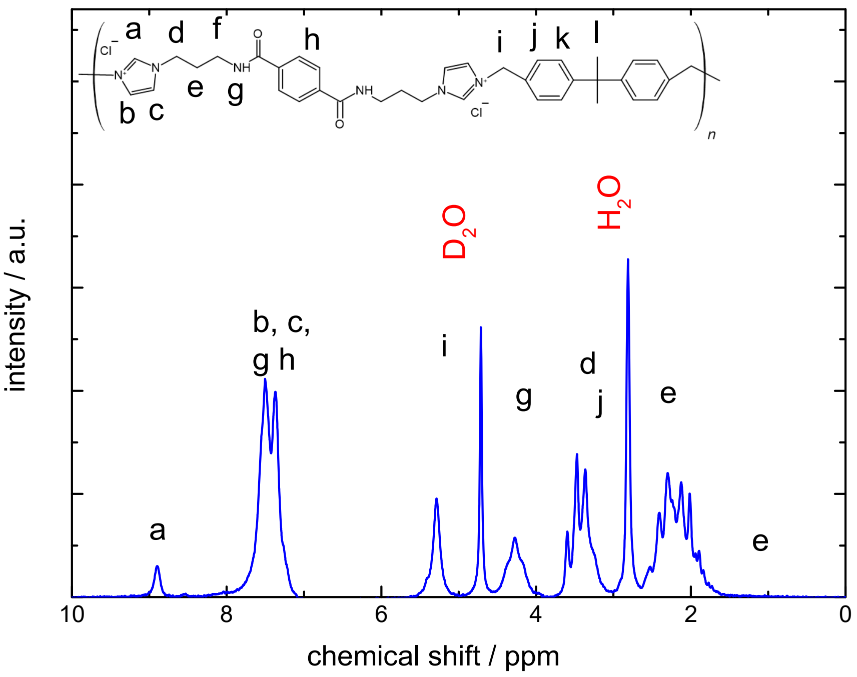

Figure S8: ^{1}H-NMR spectrum of **I4**
